# Volunteering for a better future: A pilot Sustainability Ambassadors Training Program

**DOI:** 10.1111/ajag.13069

**Published:** 2022-03-21

**Authors:** Kane Solly, Michael A. Smyer, Kim Nichols, Niklas K. Steffens, Timothy H. Kastelle, Nancy A. Pachana

**Affiliations:** ^1^ 1974 School of Psychology The University of Queensland Brisbane Queensland Australia; ^2^ 1974 Psychology Department Bucknell University Lewisburg Pennsylvania USA; ^3^ 1974 School of Education The University of Queensland Brisbane Queensland Australia; ^4^ 1974 Business School The University of Queensland Brisbane Queensland Australia

**Keywords:** ageing, climate change, empowerment, older adults, sustainability, training

## Abstract

**Objective:**

Older adults are an untapped resource for leading sustainability change. This study assessed a pilot study of a Sustainability Ambassadors Training Program for older adults.

**Methods:**

The pilot training program was conducted with a sample of 14 older adults over a single day, with some follow‐up activities for participants at home. Activities included completing a sustainable action card‐sorting activity measuring an individual's ability to make sustainable changes within their lives. A brief researcher‐devised empowerment scale was administered pre‐ and postworkshop.

**Results:**

Quantitative data revealed that sustainability empowerment increased from pre‐ to post‐training sessions and was related to the number of sustainability actions participants thought were achievable.

**Conclusions:**

The data suggest that such a brief training workshop can both yield personal change and potentially provide opportunities for a peer network to facilitate change within communities.

**Practice Impact**

With increasing research identifying the importance of meaningful activities for older people, as well as the significant impact that can have on reducing the impacts of climate change, organisations should place greater emphasis on personal change and potentially providing opportunities for peer networks to facilitate change within communities.

**Policy Impact**

This study suggests that older adults may be beneficial stakeholders in the decision‐making process around coordinating and taking effective action on climate change, as they are capable and responsive to measures that include them as partners and leaders in climate change action.

## 1 INTRODUCTION

Climate change has been defined as a quintessential intergenerational problem.^1^, ^2^ Yet, there is a pervasive negative perception that older adults do not hold pro‐environmental attitudes.^3^ This perception is contrasted against research suggesting that, as a group, older persons tend to think the same or more about the impacts of climate change than younger generations.^4^ Older adults as a group are an important source of potential solutions for environmental problems.^5^ They are respected as community leaders,^6^ with greater availability of time, resources and skills,^7^ and are highly motivated to make lasting and important contributions to society with respect to environmental volunteering.^8^

Despite this, several barriers to their engagement in sustainability efforts have been identified,^9^, ^10^ including a lack of sufficient knowledge and expertise about environmental issues, inappropriate volunteer opportunities, a lack of resources and supportive organisational structures for volunteers and a lack of socially meaningful volunteer work.

Empowerment programs are commonly adopted by environmental interventions.^11^, ^12^ Such programs aim to shift the power and focus of relationships back to the individual to act on the external forces that shape their sense of control, influence, power and capacity.^13^ Studies have shown that communities and individuals who have been empowered tend to have increased access to resources and to perpetuate this empowerment.^14^

The current research aimed to explore the effect of a pilot version of a Sustainability Ambassadors Training Program for empowering older adults to enact change within themselves, with the promise of change facilitation within their communities. This research introduces a new construct, *sustainability empowerment*, one's perceived ability to enact and create sustainability changes. It was hypothesised that self‐perceived sustainability empowerment would increase as a result of participation in the program and that empowerment would be positively related to the number of sustainable activities that participants considered achievable.

## 2 METHODS

### 2.1 Participants and study design

Participants included 14 members of Council on the Ageing (COTA) Queensland's peer educator's and ambassador's program who volunteered for this study. The sole inclusion criterion was age 50+. All participants received a small sustainability‐themed gift (e.g. keep cup or reusable green bag) in thanks for their participation.

This study was conducted using a one‐group, pre‐ and post‐test design, with pre‐ and postquestionnaires. The training day for the ambassadors was intentionally kept to a single day, based on the advice of COTA and their organisational experience of training older persons to act in similar roles (e.g. as advisors on aged care home care packages for older adults).

### 2.2 Measures

Measures were completed pre‐ and postworkshop. Participants were asked their age, sex, educational attainment, employment status, number of volunteer organisations they engaged with and the number of hours spent volunteering per week. To assess sustainability empowerment, participants completed a 9‐item scale (SEmpS) adapted from the Diabetes Empowerment Scale^15^ and the Psychological Empowerment Scale.^16^ All items were scored on a 5‐point Likert scale, anchored as *1* (*strongly disagree*) and *5* (*strongly agree*). Two example items are ‘In general, I believe that I know what helps me stay motivated to care about sustainability’ and ‘I can make a real difference in improving the sustainability of my community’. Overall scores were calculated as total average scores, with higher scores indicating higher empowerment. The scale was found to have reasonable reliability at both pretest and post‐test (*α* = 0.77 and *α* = 0.93, respectively).

During the workshop, participants completed an Australian adaptation of a sustainable action card‐sorting activity measuring an individual's ability to make sustainable changes. This was developed by Professor Michael Smyer with support from Stanford's Hasso Plattner Institute of Design. Participants sorted the 36 sustainable activities, including ‘Plant a tree’ and ‘Composting’, into four piles: ‘Things I Already Do’, ‘Things I Could Do’, ‘Things I Can't/Won't Do’ and ‘Not applicable’. These were then coded into three categories, ‘Achievable actions’ (combining ‘Things I Already Do’ with ‘Things I Could Do’), ‘Not achievable actions’ (‘Things I Can't Do’) and ‘Not applicable actions’ (‘Not Applicable’). Final scores were calculated as the total number of actions per category, which could range between 0 and 36. Higher achievable action scores reflected a greater self‐perceived capacity for making sustainable changes.

### 2.3 Procedure

Ethics approval was obtained from the University of Queensland Ethics Committee (18‐PSYCH‐4‐73‐AH).

Participants completed several tasks in small groups throughout the day (see below). The study was preregistered, and the data that support the findings of this study are openly available in OSF at https://osf.io/dy6fw/

- Introduction to the concept of sustainability; group discussion on defining sustainability as individuals.
- Small group activity on the importance of group identity and leadership strategies.
- Discussion of constructive group values that form part of positive groups.
- Sustainability card‐sorting activity (as above).
- Guest expert lecture about sustainability (e.g. using sustainable approaches to gardening, minimising plastic use and using eco‐wise appliances and homewares).
- Identification of SMART goals (Specific, Measurable, Attainable, Relevant and Time‐Bound) for sustainability.
- Instructions on home energy audit and carbon footprint calculation.
- Discussion on the importance of community engagement with sustainability.

In the month following the workshop, participants were encouraged to conduct a home energy audit, with information that had been provided during the workshop and an e‐handbook, and provided with a personal carbon footprint calculator for themselves and their family members to utilise. All participants confirmed successful completion of these activities; any questions arising from these activities or from the workshop itself were answered online. Finally, a few months after the conclusion of the workshop, participants were provided with a ‘Sustainability Calendar’, which summarised and expanded the sustainability literacy and activities covered in the workshop (e.g. eco‐friendly ways to celebrate holidays), and provided the chance to engage in activities related to sustainability over a further 12‐month period.

## 3 RESULTS

A total of 14 persons (4 men), aged between 53 and 78 years (M = 68.9 years), participated. One participant had no educational qualifications, one had completed high school, and three had a trade qualification. The rest had completed at least partial university qualifications. Half considered themselves to have retired or were undertaking occasional paid work. Nearly 80% of participants were regular volunteers at two or more organisations.

A bivariate correlation analysis was conducted. As hypothesised, higher empowerment was related to an increased number of achievable sustainable actions (pre: *r* = 0.64, *p* < 0.05; post: *r* = 0.56, *p* < 0.05), such that higher empowerment was related to more actions identified as achievable. Paired‐samples *t* tests comparing sustainability empowerment pre‐ and postprogram revealed a significant difference in scores for preprogram (M = 3.73, SD = 0.40) and postprogram empowerment (M = 4.26, SD = 0.55), t(13) = −5.52, *p* < 0.001, such that sustainability empowerment increased from pre‐ to post‐training.

## 4 DISCUSSION

Participation in the Sustainability Ambassadors Training Program was associated with greater sustainability empowerment. Older adults who scored higher on sustainability empowerment also identified more sustainability activities as achievable. This is in line with research on empowerment, as self‐efficacy has been found to mediate the relationship between empowerment and proactive behaviour in organisational leadership.^17^

This study offers preliminary support for a newly defined construct, sustainability empowerment. This is in line with previous research that has found that empowerment models are translatable into specific domains, including diabetes empowerment^18^ and empowerment in urban youth.^16^

### 4.1 Limitations

This preliminary pilot used a small sample size of individuals already well‐versed in peer‐to‐peer counselling and who expressed an interest in sustainability training. The measure of sustainability empowerment appeared to have reasonable psychometric properties but should be examined with a larger sample.

## 5 CONCLUSIONS

This study supports the further exploration of brief sustainability training programs for older adults. The program addressed the important need to engage older adults in sustainability training, empowerment and peer leadership. As the quintessential intergenerational problem of our times, climate change can only be addressed by empowering all individuals to enact change.

## CONFLICTS OF INTEREST

No conflicts of interest declared.

## References

[1] Stan I , Tom B , Richard L . The environment: the Quintessential Intergenerational Challenge. Generations. 1999;22(4):68‐71.

[2] Wunsch C , Schmitt RW , Baker DJ . Climate change as an intergenerational problem. Proc Natl Acad Sci USA. 2013;110(12):4435‐4436. 23513207 10.1073/pnas.1302536110 PMC3607060

[3] Johnson EW , Schwadel P . It is not a cohort thing: interrogating the relationship between age, cohort, and support for the environment. Environ Behav. 2019;51(7):879‐901.

[4] Kuppa S . Do Millennials See Climate Change as More Than Just a Meme? John Hopkins University; 2018.

[5] Pillemer K , Wagenet LP . Taking action: environmental volunteerism and civic engagement by older people. Public Policy Aging Rep. 2008;18(2):1‐27.

[6] Crittenden JA , DeAndrade L . Never too old to lead: activating leadership among Maine's older adults. Maine Policy Rev. 2015;24(2):80‐85.

[7] Bushway LJ , Dickinson JL , Stedman RC , Wagenet LP , Weinstein DA . Benefits, motivations, and barriers related to environmental volunteerism for older adults: developing a research agenda. Int J Aging Hum Dev. 2011;72(3):189‐206. 21834387 10.2190/AG.72.3.b

[8] Smyer MA , Pachana NA . Older adults and environmental voluntarism. In: Gu D , Dupre M , eds. Encyclopedia of Gerontology and Population Aging. Springer; 2019. doi:10.1007/978-3-319-69892-2_469-1

[9] Pillemer K , Fuller‐Rowell TE , Reid MC , Wells NM . Environmental volunteering and health outcomes over a 20‐year period. Gerontologist. 2010;50(5):594‐602. 20172902 10.1093/geront/gnq007 PMC2937248

[10] Pillemer K , Wells NM , Meador RH , Schultz L , Henderson JCR , Cope MT . Engaging older adults in environmental volunteerism: the retirees in service to the environment program. Gerontologist. 2017;57(2):367‐375. 26893490 10.1093/geront/gnv693 PMC6410889

[11] van Dop N , Depauw J , Driessens K . Measuring empowerment: development and validation of the service user psychological empowerment scale. J Soc Serv Res. 2016;42(5):651‐664.

[12] Nelson EC , Verhagen T , Noordzij ML . Health empowerment through activity trackers: an empirical smart wristband study. Comput Hum Behav. 2016;62:364‐374.

[13] Shearer NBC , Fleury J , Ward KA , O'Brien A‐M . Empowerment interventions for older adults. West J Nurs Res. 2012;34(1):24‐51. 20675621 10.1177/0193945910377887

[14] Zimmerman MA . Empowerment theory. In: Rappaport J , Seidman E , eds. Handbook of Community Psychology. Springer; 2000:43–63.

[15] Anderson RM , Funnell MM , Fitzgerald JT , Marrero DG . The Diabetes Empowerment Scale: a measure of psychosocial self‐efficacy. Diabetes Care. 2000;23(6):739‐743. 10840988 10.2337/diacare.23.6.739

[16] Ozer EJ , Schotland M . Psychological empowerment among urban youth: measure development and relationship to psychosocial functioning. Health Educ Behav. 2011;38(4):348‐356. 21606379 10.1177/1090198110373734

[17] Kim M , Beehr TA . Self‐efficacy and psychological ownership mediate the effects of empowering leadership on both good and bad employee behaviors. J Leadersh Organ Stud. 2017;24(4):466‐478.

[18] Sousa Maria R , Almeida M , Loureiro H , Martins T . Study of the psychometric properties of the Diabetes Empowerment Scale Short Form (DES‐SF). Port J Public Health. 2020;37(2–3):66‐72.

